# A pilot comparison of the retention rates of FAST and BEFAST stroke warning-sign mnemonics

**DOI:** 10.3389/fneur.2025.1624800

**Published:** 2025-07-16

**Authors:** Shujing Guo, Jeffrey L. Saver

**Affiliations:** Department of Neurology, Comprehensive Stroke Center, University of California Los Angeles, Los Angeles, CA, United States

**Keywords:** stroke, warning-sign mnemonics, BEFAST, FAST, public health education

## Abstract

**Background:**

Several pre-hospital delays prevent stroke patients from arriving within the optimal 4.5-h therapeutic window, including failure to recognize stroke symptoms and lack of urgency in perceiving them as requiring immediate medical attention. Community stroke education is critical in reducing pre-hospital delays. The Face-Arm-Speech-Time (FAST) mnemonic has been the AHA/ASA’s official stroke warning-signs public education message since 2013. Recently, a more inclusive but potentially harder to remember mnemonic, adding Balance-Eyes (BEFAST), has been proposed to increase the sensitivity of warning-sign messaging in detecting strokes. We undertook a pilot randomized trial to assess the feasibility of, refine methods for, and provide information regarding required sample size for a larger, pivotal randomized trial comparing the retention rate of FAST versus BEFAST.

**Methods:**

This study randomized adult participants without history of stroke to a comprehensive community stroke education intervention that included the definition of a stroke, its risk factors, outcomes, and either FAST or BEFAST mnemonics and assessed retention after 14–21 days. The primary endpoint was retention of mnemonic letter and warning-sign knowledge. The secondary outcome was improvement at 14–21 days in participant knowledge of stroke definition, risk factors, and outcomes.

**Results:**

Among the 50 adult participants, mean age was 37.2 (±14.8) and 30 (60%) participants were women. Study procedures were completed in all participants. At initiation, 23 (46%) participants knew the definition of stroke and could describe a mean 1.6 signs, 0.98 risk factors, and 0.74 outcomes. Full mnemonic recall rates at 14–21 days were 68% for FAST and 56% for the BEFAST cohorts (*p* = 0.39). Secondary paired analysis found significant improvements in participant knowledge of stroke signs (to 2.72), risk factors (to 3.30), and outcomes (to 2.24) with *p* = 0.007 or smaller.

**Discussion:**

This pilot trial demonstrated the feasibility of performing a large, pivotal trial and indicates a sample size of 512 participants is needed to provide sufficient power. The preliminary pilot data suggest a generally higher recall performance in the FAST group. Regardless of the mnemonic taught, participants had significant improvements in their knowledge of stroke signs, risk factors, and outcomes.

## Introduction

Acute stroke is a leading contributor to disability and mortality (1). Characterized by loss of blood flow to the brain, ischemic stroke is the most common mechanism of stroke and a highly time-sensitive emergency with a narrow therapeutic treatment window (1, 2). Estimates based on advanced neuroimaging indicate that ischemic stroke patients lose 1.9 million neurons per minute of delayed treatment (3). Therapeutic interventions are most effective when administered as soon as possible after symptom onset, with the maximal benefit within the first hour after last known well time, often referred to as the “golden hour.” (4) Current thrombolytic therapies have extended the therapeutic window up to 4.5 h after onset (5). Yet, less than half of patients arrive within the 4.5 h window and studies have shown an uptrend in delayed presentation throughout the past decade (6, 7). The delay is largely due to patient and witness pre-hospital factors, including failure to recognize stroke symptoms and to identify them as requiring immediate medical attention (8–11).

Community stroke education is a critical factor in reducing pre-hospital delays. Stroke education campaigns serve to increase awareness of stroke symptoms, reducing the time from onset to emergency department (ED) arrival (10, 12–14). Past studies have shown that education campaigns emphasizing the urgent nature of strokes encourages individuals to seek emergency medical help faster (9, 14, 15).

In the United States, public health messaging by the American Heart/Stroke Association (AHA/ASA) and other national and regional organizations to educate lay individuals on stroke warning signs and the importance of immediately activating the emergency medical system have evolved over time. In the initial era of proven acute stroke therapy, from 1996 to 2012, public health education campaigns focused upon educating the public on the “Five Suddens” (sudden confusion, sudden trouble speaking, sudden numbness or weakness, sudden severe headache, and sudden visual trouble) (16). In 2008, the Stroke Heroes Act FAST study, performed by the Massachusetts Department of Public Health, determined that a shorter, four-item, mnemonic-focused campaign could be highly effective in improving community stroke knowledge (17). As a result, in 2013, the FAST mnemonic, based on the Cincinnati Prehospital Stroke Scale, was formally adopted as the AHA/ASA national public education stroke warning sign message (16). FAST stands for: Face–Arm–Speech–Time, instructing lay individuals to look for any facial droop (F), arm drift (A), or speech difficulty (S) as a sign of potential acute stroke. If any such symptoms are present, individuals are instructed it is time (T) to call 911 and activate emergency medical services immediately. The FAST mnemonic remains the official AHA/ASA message in 2025, but has been criticized for lack of comprehensiveness. While it captures weakness and language abnormality which are the two most common and most debilitating of stroke signs, it does not include other important symptoms (18). To address this limitation, the BEFAST mnemonic was devised, adding two additional recognition signs to FAST: Balance (B) and Eyes (E), instructing lay individuals to also look for any gait imbalance/vertigo or any change in vision or eye movement (19). The BEFAST mnemonic has been validated as a sensitive screening tool for acute ischemic stroke patients (20).

The BEFAST and FAST mnemonics have contrasting advantages and disadvantages. BEFAST enables recognition of strokes that present with B-E but not F-A-S symptoms, providing a more comprehensive understanding of stroke warning signs (21, 22). There is evidence that the inclusion of B-E symptoms could theoretically increase detection of posterior circulation strokes compared with F-A-S alone from 85.9 to 95.6% (19); however, BEFAST also increases false positives, potentially increasing Emergency Department and stroke team work burden (23). Rural and smaller hospitals with limited resources are particularly susceptible to being overwhelmed by such false positives. In addition, it is possible that the longer, six-element BEFAST mnemonic is more difficult to remember than the simpler, four-element FAST mnemonic. Formal cognitive psychology studies have demonstrated that human memory is optimal up to a capacity limit of four and then degrades as more items are added (24). Accordingly, it is possible the longer BEFAST mnemonic could paradoxically produce less, not more, information retention after exposure. The comparative retention of the FAST and BEFAST acronyms by members of the general public has not been well delineated.

We therefore undertook a pilot randomized trial to assess the feasibility of, and inform sample size calculations for, a large, pivotal trial comparing the retention rate of FAST versus BEFAST among adults using a comprehensive, short community stroke education paradigm with a delayed recall assessment.

## Methods

The present study was a randomized pilot trial that recruited 50 adult volunteers. Eligibility criteria were: (1) Age ≥ 25 years; (2) No personal medical history of stroke; and (3) English-speaking. The study was certified exempt by the institutional review board at UCLA as a minimal risk study.

A convenience sample of individuals was recruited from both The Church in Los Angeles and the Ronald Reagan Medical Center (RRMC) UCLA Neurology Faculty Clinic. The Church in Los Angeles is a group of five churches throughout the Los Angeles County area with a diverse membership comprising of many different ethnicities, socioeconomic statuses, ages, and occupations. The opportunity to participate in the study was communicated via flyers, text messaging, and a study announcement in the weekly church newsletter. Recruitment materials included either a QR code or a direct link to a Calendly page. Interested participants self-screened for eligibility and booked the first study meeting through Calendly. Participants then received an email with a unique link to their scheduled, private Zoom teleconference. At the first meeting, the investigator (SG) confirmed participant eligibility, provided participants with an overview of study procedures, and elicited oral informed consent. Participants received $20 compensation for the time they took to participate.

After providing consent, participant baseline knowledge of stroke pathophysiology and stroke warning signs were assessed. Participants were asked to: (1) indicate what a stroke was; (2) list any stroke warning signs or symptoms of which they were aware; and (3) state any other stroke information they may already know.

Participants were then randomly assigned, in a 1:1 ratio, into the FAST education group or the BEFAST education group using a permuted block randomization program (block size of four without strata). Each cohort had 25 participants.

**Figure 1:**
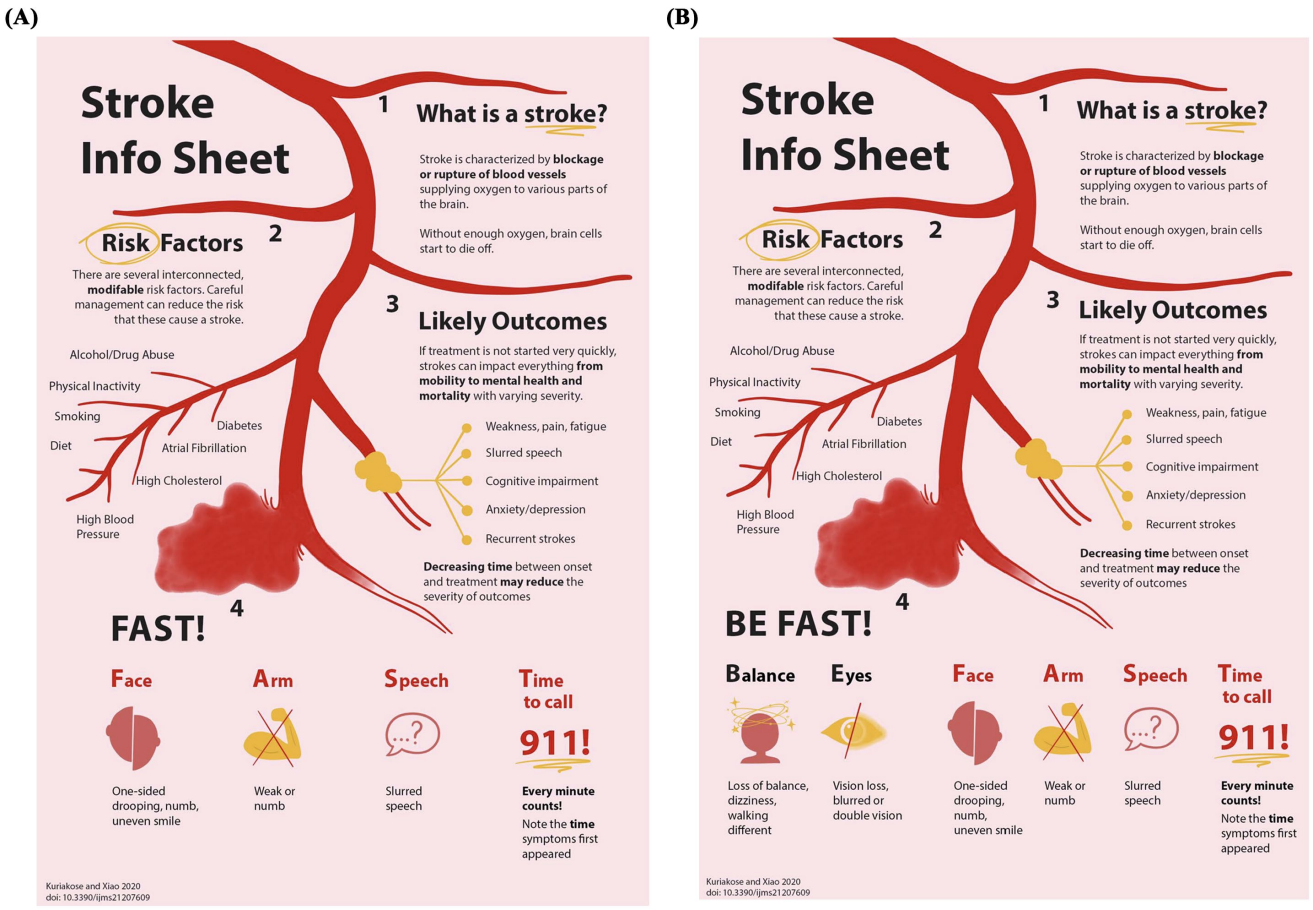
Educational intervention infographics. Participants were educated using these combined text and figure educational information sheets. The top three-quarters of each sheet provided identical presentations of what a stroke is, stroke risk factors, and stroke outcomes. The bottom quarter of each sheet showed the warning sign mnemonic to which the participant had been randomly allocated: **(A)** FAST, **(B)** BEFAST.

After allocation, participants were presented with an educational infographic delineating the definition of stroke, its risk factors, outcomes, and recognition mnemonics. The infographic presented to each group was identical except for the mnemonic displayed, which was aligned with their randomization assignment (Figure 1). The information on the infographic was presented both visually and auditorily. While the participant viewed the infographic page, the investigator (SG) read aloud the information on the infographic section by section. The lists of risk factors, outcomes, and symptoms were strung together into coherent sentence. The term “Atrial Fibrillation” was immediately preceded with “heart conditions such as [atrial fibrillation].”

The information retention visit was scheduled to occur 14–21 days after the educational session. During this time, the protocol did not mandate rehearsal of the presented information. At the follow-up visit, participants were asked to: (1) recall the letters of respective acronym; and (2) recall the symptoms/signs associated with each letter. Subsequently, participants were asked to recall any additional information about: (3) list any other stroke warning signs or symptoms of which they were aware; (4) what a stroke was, (5) its risk factors, and (6) its outcomes.

### Outcomes

The primary outcome was the proportion of participants completing all study procedures. Feasibility of completing a large, pivotal trial using similar procedures would be considered demonstrated if ≥90% of participants fully completed the protocol. A lower retention rate would be considered as indicating a potential need to alter study procedures to improve participant protocol completion. An additional non-numeric feasibility goal was to refine and optimize the methods of study conduct.

The lead secondary outcome was retention of the complete mnemonic acronym. Secondary mnemonic recall outcomes were: (1) retention of individual acronym letters and their associated words; and (2) retention of the signs/symptoms encompassed by each component of the acronym (even if the acronym letter and word for that symptom/sign was not recalled). Full acronym recall was assessed as a dichotomous Yes/No result. Detailed recall performance was analyzed by summing the letter component (the letter and its associated word) and individual sign/symptom component (the signs associated with each letter item).

Participants’ recall performance for each element was encoded as CLCS, correct letter and correct sign; CLIS, correct letter but incorrect sign; CL, correct letter only; CS, correct sign only; or N, none recalled. Total letter recall contained all participants in each group that recalled the letter, regardless of whether the corresponding sign was recalled (CLCS + CLIS + CL). Total sign recall contained all participants in each group that recalled the sign, regardless of whether the corresponding letter was recalled (CLCS + CS).

Additional secondary outcomes evaluated improvement in participants’ knowledge of stroke facts (including definition, signs/symptoms, risk factors, and outcomes) at baseline compared to recall. We also examined which categories of stroke knowledge demonstrated the greatest improvement after educational intervention.

### Statistical analysis

Participant demographic features were characterized with descriptive statistics, including mean and standard deviation for continuous variables (age) and participant number and percentages for binary and categorical variables (sex, highest education degree, occupation). Participant characteristics in the FAST and the BEFAST groups were compared using t-tests for continuous measures, Fisher’s Exact Test for binary measures, and chi-square for categorical measures with multiple response options.

The sample size for this pilot study was selected based on the feasibility assessment objective. Based on published recommendations for feasibility study size and the senior author’s past experience, a sample size of 50 participants (25 per group) was deemed adequate (25).

For the secondary outcomes assessing change/improvement in stroke knowledge between pre-intervention baseline and the 14–21-day follow-up visit in the FAST versus BEFAST groups, we performed paired t-tests for paired continuous measures, Fisher’s Exact Test for binary measures of differences across sessions, and chi-square for multicategory measures of difference across sessions. The analysis of between group differences in retention of individual mnemonic letters and symptom-signs was performed for the four F-A-S-T elements as these are shared between the groups and they index the most common stroke symptoms/signs. All *p* values are two-sided.

## Results

All 50 participants were enrolled in November 2024 and completed both study visits between November and December 2024. Two potential participants declined to begin participation in the study after the study investigator explained the study procedures. All other potential participants orally consented to the study. Participant characteristics are presented in Table 1. Across the entire study group, mean age was 37.2 (±14.8) and 30 (60%) participants were women. The educational background of participants included 1 (2%) high school diploma, 34 (68%) bachelor’s degrees, 7 (14%) master’s degrees, and 8 (16%) doctorate degrees. Within the study group, 3 (6%) of participants were unemployed or stay at home parents. Of the top three most common occupations, 10 (20%) were students, 9 (18%) were campus ministers, and 4 (8%) were research scientists. The time interval from educational exposure to recall assessment was 16.5 (±3.1) days. These characteristics were similar in the FAST and BEFAST education groups.

**Table 1:** Participant characteristics.

Characteristic	FAST	BEFAST
	(*n* = 25)	(*n* = 25)
Age, mean (SD)	37.2 (12.8)	37.1 (16.2)
Sex, female, *n* (%)	13 (37)	17 (33)
Education, *n* (%)		
High Schools	1 (4)	0 (0)
Bachelors	14 (56)	20 (80)
Masters	4 (16)	3 (2)
PhD	6 (24)	2 (8)
Occupation, *n* (%)		
Student	3 (12)	7 (28)
Employed	20 (80)	17 (68)
Unemployed	2 (8)	1 (4)
Interval until recall (days), mean (SD)	17.0 (3.1)	16.1 (2.9)

FAST, Face–Arm–Speech–Time; BEFAST, Balance–Eyes–Face–Arm–Speech–Time; HS, high school; SD, standard deviation.

At the initial visit, prior to the educational intervention, baseline knowledge was assessed (Table 2). Among the 50 participants, 23 (46%) participants knew at least one key aspect of the definition of stroke. Of those, 61% (*n* = 14) described an ischemic stroke including the occurrence of blocking of a blood vessel, 4% (*n* = 1) described a hemorrhagic stroke, 13% (*n* = 3) described stroke using both ischemic and hemorrhagic definitions, and 22% (*n* = 5) gave an approximate, partially accurate description of stroke, e.g., as reduced blood flow or oxygen to the brain without mention of a blocked blood vessel. A total of 36 (72%) participants were able to report at least one symptom/sign of stroke (mean 1.6) while 14 (28%) reported none. The three most commonly reported symptoms and signs were: (1) paralysis (36%); (2) speech (24%); (3) asymmetry (22%). For stroke risk factors, 20 (40%) of participants were able to mention at least one (mean 0.98) while 30 (60%) reported none. The three most commonly reported risk factors were: (1) diet (12%); (2) high blood pressure (12%); (3) age (10%). For stroke outcomes, 19 (38%) of participants were able to mention at least one (mean 0.74) while 31 (62%) reported none. The three most commonly reported stroke outcomes were: (1) paralysis (18%); (2) death (10%); (3) functional impairments (8%).

**Table 2:** Baseline knowledge.

Knowledge category	All participants
	(*n* = 50)
Definition, *n* (%)	
Ischemic	14 (61)
Hemorrhagic	1 (4)
Ischemic and Hemorrhagic	3 (13)
Approximate^*^	5 (22)
None	27 (54)
Signs/Symptoms, mean (SD)	1.60 (1.44)
≥1 item, *n* (%)	36 (72)
None	14 (28)
Risk Factors, mean (SD)	0.98 (1.56)
≥1 item, *n* (%)	20 (40)
None	30 (60)
Outcomes, mean (SD)	0.74 (1.16)
≥1 item, *n* (%)	19 (38)
None	31 (62)

^*^Approximate answers were partially accurate, e.g., description of stroke as reduced blood flow or oxygen to the brain without mention of a blocked blood vessel.

### Primary outcome

All 50 participants (100%) completed the full study protocol. There was no data missingness for any study item.

### Additional feasibility goal—study conduct refinement

Several aspects of study conduct were refined or validated over the course of the pilot period, including:

- When asked initial open-ended questions regarding what warning signs, risk factors, and outcomes they recalled, participants would often provide somewhat extended responses that could include paraphrased versions of the target items. Writing down all that they said enabled later careful analysis of whether the semantic content of the target item had been recalled.
- Follow-up questions requesting specifics within a broad category can surface retained granular knowledge. For example, when a patient stated that the broad category of “heart conditions” was a risk factor for stroke, a follow-up asking which specific heart conditions could elicit the target item of atrial fibrillation.
- Spending the last few minutes of the first teaching session scheduling the exact date and time of the subsequent retention assessment session worked well in yielding a high follow-up rate.

### Lead secondary outcome

At the delayed recall visit, full mnemonic recall was achieved by 68% of the FAST and 56% of the BEFAST cohorts (*p* = 0.39 by Fisher’s Exact Test).

**Figure 2:**
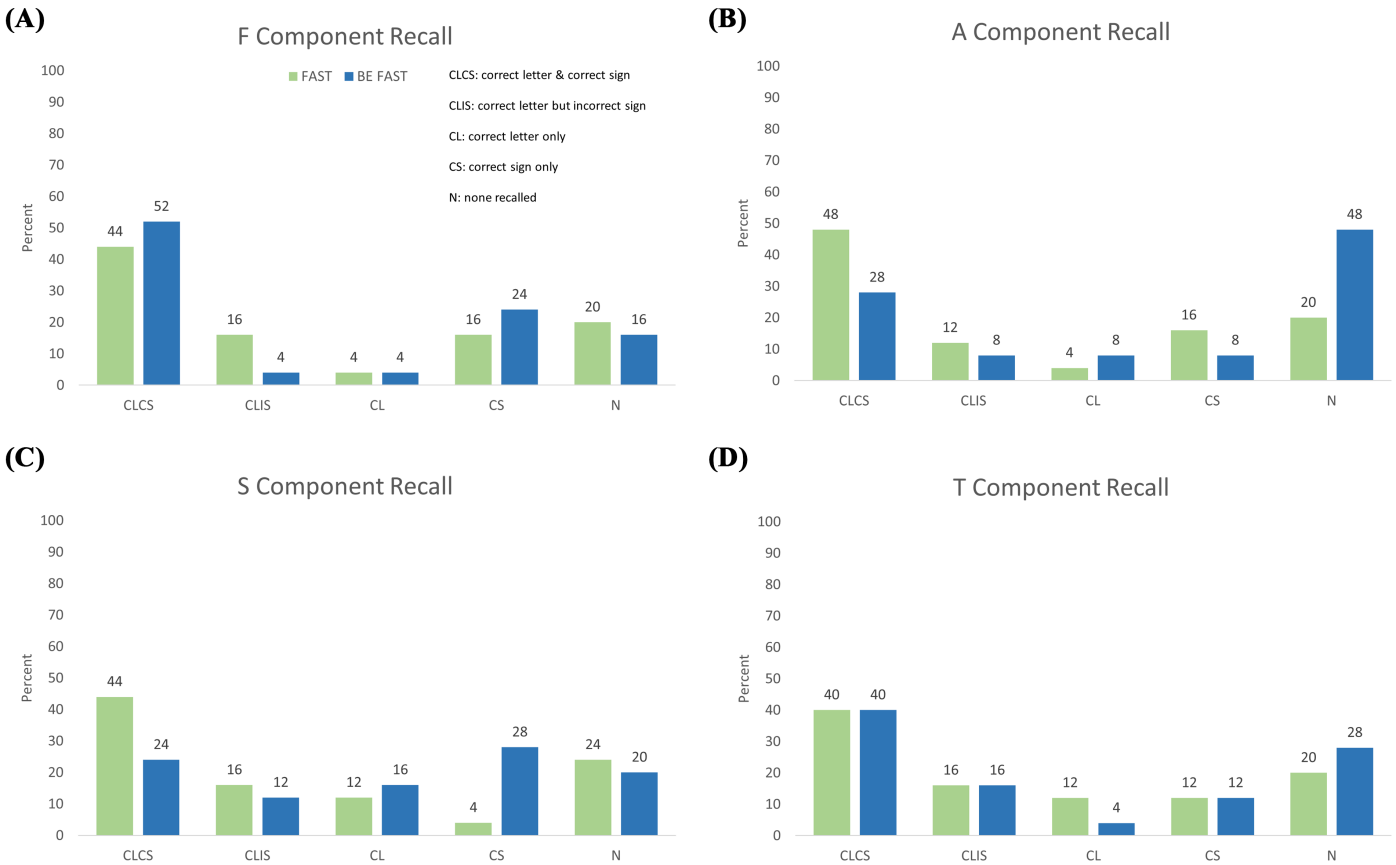
Granular recall performance of F-A-S-T elements. Frequency of different recall success for the four FAST items among participants randomly allocated to FAST training (green) or BEFAST training (blue). CLCS, both letter correct and sign correct. of individual shared mnemonic components broken down by successful recall of both the correct mnemonic letter and its corresponding sign/symptom (CLCS), the correct letter but with an incorrectly associated sign (CLIS), only the correct letter (CL), only the correct sign (CS), or no correct recall of either letter or sign (N). Panels display the percentage of each recall combination for **(A)** the F component, **(B)** A component, **(C)** S component, and **(D)** T component.

Recall for the individual F-A-S-T elements contained in both mnemonics is shown in Table 3 and Figure 2. For the F-Face element (Figure 2A), 64% of FAST and 60% of BEFAST participants recalled the letter (*p* = 1.00 by Fisher’s Exact Test) while 60 and 75%, respectively, recalled the corresponding sign (*p* = 0.36). The A-Arm element (Figure 2B) saw 64 and 44% letter recall (*p* = 0.26) and 64 and 36% sign recall (*p* = 0.09). The S-Speech element (Figure 2C) had 72 and 52% letter recall (*p* = 0.24) and 48 and 52% sign recall (*p* = 1.00). The T-Time element (Figure 2D) had 68 and 60% letter recall (*p* = 0.77) and 52 and 52% sign recall (*p* = 1.00). There was also no statistically significant difference in total letter recall and total sign recall between the two groups (*p* = 0.51). When recall frequencies for F-A-S-T elements are combined, the FAST group recalled the letter 67 times and the correct sign 56 times. The BEFAST group recalled the letter and correct sign 54 times each.

**Table 3:** Retention of individual component letters/symptoms-signs comprising mnemonics at 14–21 days.

Item being recalled^*^	FAST	BEFAST
	(*n* = 25)	(*n* = 25)
Balance (%)		
CLCS: Letter and Sign		8 (32)
CLIS: Incorrect Sign		2 (8)
LC: Letter Only		2 (8)
SC: Sign Only		4 (16)
N: None		9 (36)
Eyes (%)		
CLCS		7 (28)
CLIS		1 (4)
CL		5 (20)
CS		2 (8)
N		10 (40)
Face (%)		
CLCS	11 (44)	13 (52)
CLIS	4 (16)	1 (4)
CL	1 (4)	1 (4)
CS	4 (16)	6 (24)
N	5 (20)	4 (16)
Arm (%)		
CLCS	12 (48)	7 (28)
CLIS	3 (12)	2 (8)
CL	1 (4)	2 (8)
CS	4 (16)	2 (8)
N	5 (20)	12 (48)
Speech (%)		
CLCS	11 (44)	6 (24)
CLIS	4 (16)	3 (12)
CL	3 (12)	4 (16)
CS	1 (4)	7 (28)
N	6 (24)	5 (20)
Time (%)		
CLCS	10 (40)	10 (40)
CLIS	4 (16)	4 (16)
CL	3 (12)	1 (4)
CS	3 (12)	3 (12)
N	5 (20)	7 (28)

^*^CLCS, correct letter and correct sign; CLIS, correct letter but incorrect sign; CL, correct letter only; CS, correct sign only; N, neither recalled.

### Additional secondary outcomes

**Figure 3:**
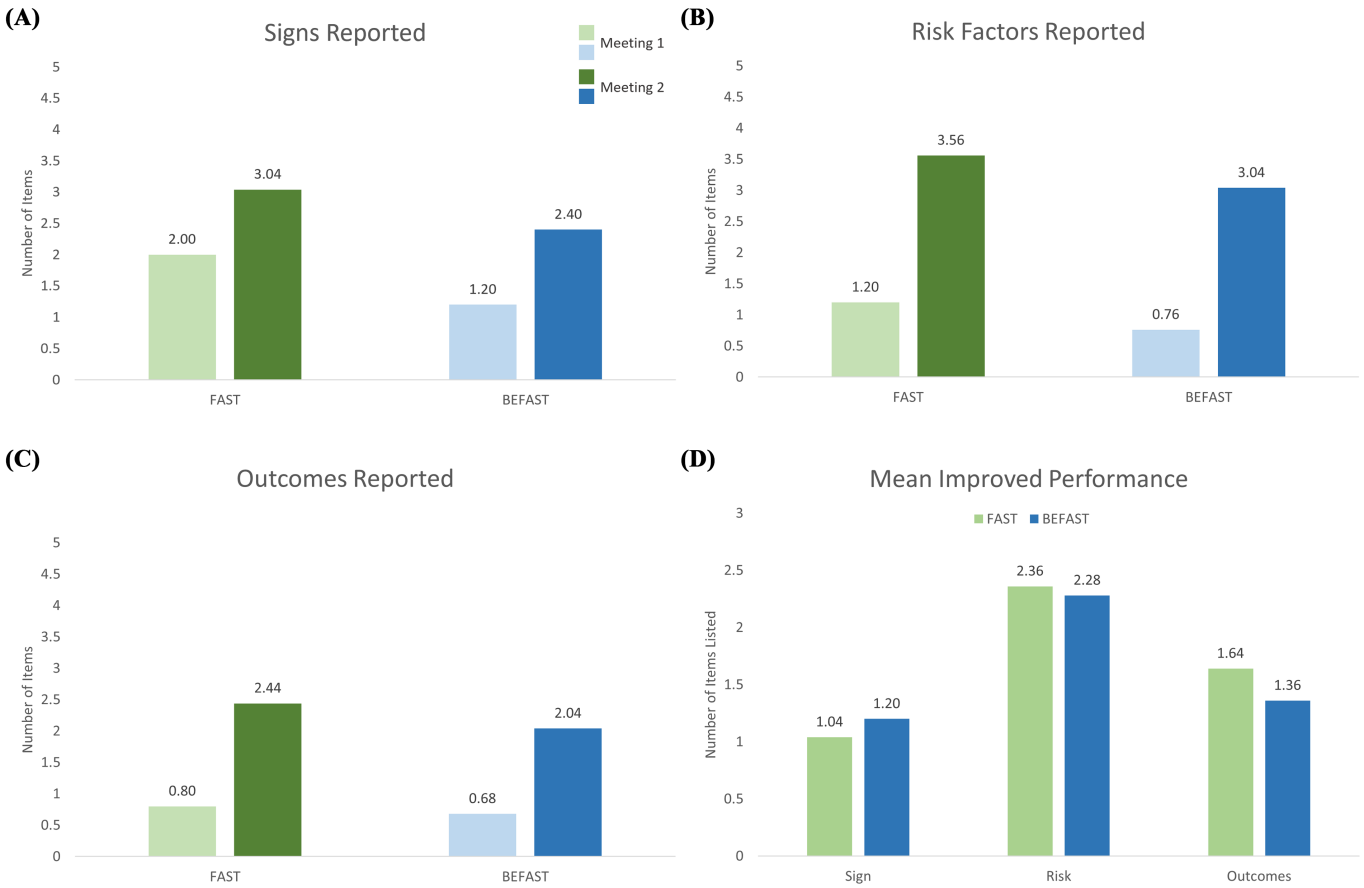
Improvement from pre-intervention baseline to delayed retention assessment in stroke knowledge in the FAST (blue) and BEFAST (green) exposure groups. **(A)** Number of stroke signs/symptoms reported pre-intervention and at delayed recall. **(B)** Number of stroke risk factors reported pre-intervention and at delayed recall. **(C)** Number of stroke outcomes reported pre-intervention and at delayed recall. **(D)** Mean increase in items recalled for: F-A-S stroke signs/symptoms (among 3 items), stroke risk factors (among 8 items), and stroke outcomes (among 5 items). Both exposure groups showed substantial retained improvement in all three categories.

Both groups showed improvements in each of the stroke knowledge categories of symptom/sign, risk factors, and outcomes. Figures 3A–C shows the mean number of items reported in each category by each educational group in Meeting 1 and in Meeting 2. The FAST group reported a mean of 2.0 (±1.5) signs in Meeting 1 and a mean of 3.0 (±1.3) signs in Meeting 2 (*p* = 0.007 by paired t-test). Similarly, the BEFAST group reported a mean of 1.2 (±1.3) signs in Meeting 1 and 2.4 (±1.0) in Meeting 2 (*p* < 0.0001). The FAST group reported 1.2 (±1.9) risk factors at baseline and 3.6 (±2.31) at recall assessment (*p* < 0.0001). The BEFAST group reported 0.8 (±1.2) and 3.0 (±2.1) risk factors in Meeting 1 and Meeting 2, respectively (*p* < 0.0001). The FAST group mentioned a mean 0.8 (±1.2) outcomes in Meeting 1 and 2.4 (±1.9) outcomes in Meeting 2 (*p* = 0.0005). BEFAST participants reported a mean 0.7 (±1.1) and 2.0 (±1.5) outcomes in Meeting 1 and Meeting 2, respectively (*p* = 0.001). There was no statistically significant difference in prior stroke knowledge, at baseline, between the FAST and BEFAST groups (*p* = 0.98).

Figure 3D depicts the mean change from pre-intervention baseline to delayed retention assessment in the number of items reported for each of the three knowledge categories between the FAST and BEFAST groups: F-A-S stroke signs/symptoms, risk factors, and outcomes.

## Discussion

This study demonstrated the feasibility of performing a randomized trial to examine FAST and BEFAST mnemonic retention in the context of a comprehensive stroke education paradigm. The brief education program was successfully delivered to all participants and all completed the follow-up assessment. Granular collected study data enabled analysis of several aspects of interest, including the retention of acronyms, retention of meaning of acronym letters, retention of the stroke signs with or without acronym association, and changes in knowledge regarding stroke pathophysiology, risk factors, and outcomes.

In addition, the study enabled investigators to assess and refine or validate several methods of study conduct. Detailed aspects of interviewer interaction with participants were improved and the approach to study calendaring successfully pressure-tested. The end-of-pilot study methods provide a robust basis for successful conduct of a successor pivotal trial.

The mnemonic-anchored comprehensive education program used in this study is informed by educational principles. Multiple studies have demonstrated that when mnemonics are incorporated to learning material, recall of the material is better than when mnemonics are not used. Both FAST and BEFAST are “encoding mnemonics,” designed to allow learners to transform new, abstract information into concrete, easily memorable information. They are also acronymic mnemonics, memory aids in which the first letters of a list of items are combined to form a word. The new word then serves as a reminder of the original list, condensing the information into a more easily remembered form. This study also implements educational best practice by performing a randomized trial comparing two educational interventions. An extensive literature recognizes that evidence from randomized field trials are an indispensable source of high-quality information to guide educational policies (26). We selected a conservative approach to letter recall analysis. This approach required participants to list the mnemonic letter along with its associated word for the letter recall attempt to be considered successful. For example, “A” must be recalled as “A for Arm” to be considered a successful recall attempt. In contrast to a more liberal approach where simply the letter “A” would qualify as successful recall, the conservative approach guards against counting incorrect letter and mnemonic word pairs such as “A for Amnesia” as successful recall of the mnemonic.

As a pilot trial, this study was underpowered to test the underlying hypothesis that the FAST group would demonstrate higher retention of key warning sign elements than the BEFAST group. As expected, within this study there was no statistically significant difference in recall performance for the full mnemonic, letter components, or sign components. Nevertheless, the directionality of differences in estimands in our primary mnemonic component recall outcome align with the result of a larger randomized trial published after the inception of the current study. In that 174-participant randomized trial investigating FAST versus BEFAST, retention was statistically significantly better for the FAST mnemonic (27). Both studies provide indications that the longer BEFAST mnemonic overtaxes the human memory system while the four-item FAST aligns with the limits of human working memory capacity that processes briefly presented public messaging (24).

The current study had a more comprehensive aspect than the recent, larger trial, as it assessed retention of education about additional aspects of stroke than just mnemonic components and meaning. This study therefore provides unique data regarding the effectiveness of embedding general stroke knowledge messages within a warning signs mnemonic-based education intervention. The study identified a statistically significant benefit to a comprehensive stroke education intervention in the recall of stroke definition and three knowledge categories: 1) signs/symptoms apart from mnemonic recall, 2) risks factors, and 3) outcomes. Between the educational exposure and the recall sessions, participants in both the FAST and BEFAST education arms were able to state more than double the number of items in all three categories at the recall session. Further studies of larger, more representative samples are desirable to confirm whether there is generally a greater increase in the retention of stroke risk factors, outcomes, and signs when educational interventions are or are not warning signs mnemonic-based.

Among these additional topic areas, signs and symptoms knowledge exhibited the smallest improvement while the greatest increase was observed in the risk factors category where both groups approximately tripled recall values. This substantial increase in risk factor knowledge may in part be attributed to the inclusion of only modifiable risk factors in the educational intervention. During the educational meeting, it was emphasized that these risk factors are controllable through lifestyle choices, aligning with participant interest in healthy lifestyle choices that would prevent a stroke.

This study has limitations. First, the modest sample size was appropriate for a pilot trial but underpowered to test the underlying hypothesis of superiority of the FAST over the BEFAST mnemonic. The sample size of 50 participants had sufficient power to detect superiority only if there were very large success rate differences between the mnemonics, 35% versus 72%. By contrast, the 68% versus 56% difference found in this study would require a sample size of 512 (256 in each group) study completers to be sufficiently powered (and recruitment and retention rates in a larger, more heterogenous sample may be lower than in the current study). Nonetheless, the results of this pilot trial do align with a concurrently performed larger trial. Second, in the convenience sampling, all participants were a single church congregation. Although there was a variety of ethnicities, occupations, and educational degrees within both groups, they were similar to each other in other sociocultural respects. The group also had more individuals with higher education than a general population. Future investigations in more diverse populations are needed. Third, the age of participants was younger than the age of typical stroke patients. However, recognition of stroke and activation of the emergency medical system is often performed by family members and other witnesses rather than patients themselves, so this is an important target group. Fourth, at baseline the FAST group had nonsignificantly more stroke knowledge than the BEFAST group. This could have aided their learning by providing a stronger starting foundation or limited their learning by providing a ceiling effect. Fifth, this study assessed delayed recall at 2 weeks and the recent larger trial assessed at 1 month. The durability of retention over longer time periods merits investigation.

In conclusion, this study successfully demonstrated the feasibility of a randomized investigation comparing FAST and BEFAST mnemonic retention within a comprehensive stroke education paradigm. The preliminary pilot data suggest a potentially higher recall performance in the FAST group, consistent with a contemporaneous study, and provides key information for pivotal trial sample size planning. Embedding a more comprehensive stroke education module within the mnemonic-based, warning sign education paradigm was associated with improvements in participant knowledge of stroke signs, risk factors, and outcomes. The combination of a galvanizing message regarding stroke warning signs with broader information about cerebrovascular disease may be a beneficial strategy for public education about stroke.

## Data Availability

The raw data supporting the conclusions of this article will be made available by the authors, without undue reservation.
